# NO_2_ and NH_3_ Sensing Characteristics of Inkjet Printing Graphene Gas Sensors

**DOI:** 10.3390/s19153379

**Published:** 2019-08-01

**Authors:** Caterina Travan, Alexander Bergmann

**Affiliations:** 1 Infineon Technology AG, 85579 Neubiberg, Germany; 2 Institute of Electronic Sensor Systems, Technische Universität Graz, 8010 Graz, Austria

**Keywords:** graphene, inkjet printing, gas sensor

## Abstract

Graphene is a good candidate for filling the market requirements for cheap, high sensitivity, robust towards contamination, low noise, and low power consumption gas sensors, thanks to its unique properties, i.e., large surface, high mobility, and long-term stability. Inkjet printing is a cheap additive manufacturing method allowing fast, relatively precise and contactless deposition of a wide range of materials; it can be considered therefore the ideal technique for fast deposition of graphene films on thin substrates. In this paper, the sensitivity of graphene-based chemiresistor gas sensors, fabricated through inkjet printing, is investigated using different concentrations of graphene in the inks. Samples have been produced and characterized in terms of response towards humidity, nitrogen dioxide, and ammonia. The presented results highlight the importance of tuning the layer thickness and achieving good film homogeneity in order to maximize the sensitivity of the sensor.

## 1. Introduction

Good breathing air quality is one of the most important factors for a healthy life. Increasing consciousness within the population and institutions is translating into a substantial increase in the demand for cheap, low power, high sensitivity, and reliable gas sensors for monitoring the concentration of pollutants in indoor and outdoor environments. Gas sensors partially filling these market’s requirements are MOX (metal oxide) or polymer based chemiresistor gas sensors [1,2,3]. However, such sensors present several disadvantages: They require high operating temperatures (up to 600 °C) due to slow desorption characteristics, are sensitive towards humidity and show baseline drift, which require frequent re-calibration [2,4,5,6]. A better alternative might be represented by the use of graphene-based electrochemical gas sensors. Graphene offers indeed high sensitivity and fast response time thanks to its two-dimensional structure and low noise due to its high conductivity [7,8,9,10,11,12]. However, mass manufacturing this type of sensor still presents major challenges. The use of graphene produced by CVD (chemical vapor deposition), which is widely found in literature [13], is not applicable due to high related costs. The production of such sensors requires a direct writing technique such as inkjet printing [14,15,16]. Inkjet printing is a cheap additive manufacturing technique allowing fast, relatively precise and contact-less deposition of a wide range of materials [17,18,19,20]. The creation of a homogeneous film with constant thickness using such technique is challenging due to the tolerance of the nominal droplet volume and the accumulation of material at the edge of the printed structure, due to the evaporation gradient, so-called coffee ring effect [21]. Most of the graphene inks found in literature are either water-based or NMP (N-Methyl-2-pyrrolidon)-based inks [22,23,24]. Water-based inks are widely used in research but the process is not scalable to mass production. Productive inkjet printers’ printheads are indeed not suitable for water-based inks due to the materials employed for the nozzle plate, moreover NMP (N-Methyl-2-pyrrolidon) is not desired as a solvent due to its toxicity and corrosiveness of the gases it releases [25], which would damage the printhead and potentially other mechanical parts of the printer, e.g., the *x*, *y*, *z* stage.

This paper is structured as follows: Section 2 introduces the materials, measurement techniques, and equipment used to carry out the experiments; Section 3 describes the steps carried out during the investigation: An analytical study of the samples is first presented. Afterwards, gas measurements are carried out and results correlated with the previous analysis. Finally, Section 4 summarizes the results and draws the conclusions.

## 2. Materials and Methods

The ink used in this work was developed by an external partner, to meet the requirements of the employed printing hardware. The exact composition of the ink was not disclosed but the ink uses terpineol as the main solvent and few-layers graphene flakes with very low oxygen content. Due to confidentially agreements, more information on the ink may not be disclosed. For more information on the ink you may contact caterina.travan@infineon.com. The ink was characterized in terms of density, viscosity, and surface tension at Joanneum Research in Weiz in order to assess its compatibility with the printhead used and to calculate its Ohnesorge numbers before testing its jetting performance. The density of the ink was measured using a volume measuring container and a scale. The rheological measurements were performed with a rotational viscometer Brookfield DV-III Ultra. The surface tension was measured with the pendant drop method using Krüss DSA 100, a drop shape analyzer produced by Krüss. The employed ink had a density of about 1 g/cm^3^, a viscosity between 8 and 14 cP (at 25 °C and using a shear rate of 500 s^−1^), and a total surface energy between 30 and 35 mN/m; it therefore showed compatible rheological characteristics for optimum jetting performance and printing results, according to the dimensionless figure of merit, used to characterized the drop behavior in the DOD (Drop On Demand) printing technology [15,18,26]. The thickness of the flakes used for this work is in the order of few-layers graphene, which have a lateral dimension below 0.5 µm.

Raman spectroscopy was used to investigate the amount of defects in the graphene flakes present in the ink. Raman spectra were measured at CTR (Carinthian Tech Research) in Villach using a Renishaw inVia Reflex Raman spectroscope. The spectra were acquired using a laser excitation of 532 nm, an aperture of 50%, and an exposure time of 1 s. The obtained laser spot had a size of about 5 µm. The samples were kept at room temperature during the measurements. A Raman analysis was performed in combination with an optical investigation using SEM, which provided information about lateral flake size and flake distribution. SEM pictures were taken using an Ultra-High-Resolution HitachiSU-8010. After verifying the good droplet formation and jetting performance, the ink was printed on gold interdigitated electrodes (IDEs) on a silicon substrate with an isolating silicon nitride layer between the IDEs and the silicon. All the jetting and printing trials were carried out using a LP50 printer, an R&D (Research and Development) DOD piezoelectric printer produced by Meyer Burger. The printheads used in this work are SE128AA produced by FUJIFILM Dimatix, Inc. After printing, samples were thermally treated in IR vacuum reflow oven with a single process chamber SRO-700, produced by ATV, in order to remove residues of solvent and burn off stabilizers and surfactants present in the ink.

**Figure 1:**
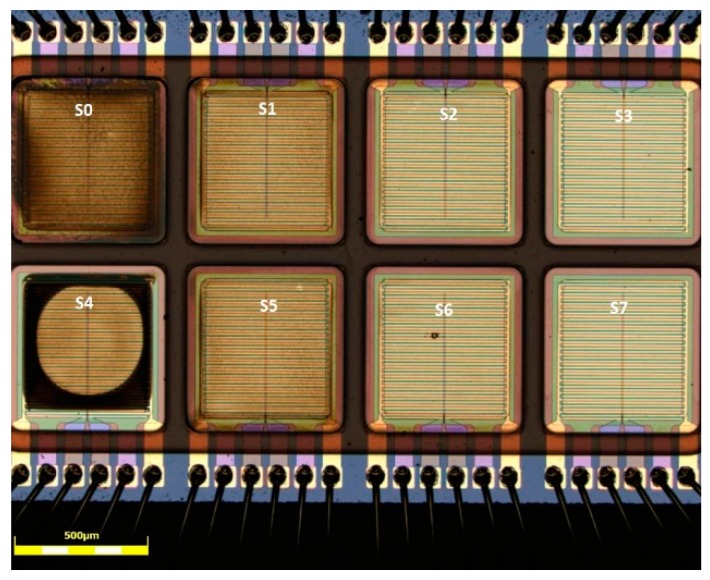
Optical microscope picture of the 8 sensor fields after printing and drying.

In order to investigate the influence of the graphene concentration on the sensitivity towards NO_2_ and NH_3,_ inks, the following graphene concentrations were used: 5 g/L (sensor S_0_ and S_4_), 2.5 g/L (sensor S_1_ and S_5_), 1 g/L (sensor S_2_ and S_6_), 0.5 g/L (sensor S_3_ and S_7_) as shown in Figure 1. All these inks were printed keeping the number of droplets deposited on each electrode constant. A printing resolution of 750 dpi × 1000 dpi and a print file of 200 µm × 480 µm were used. Different thickness in the graphene layer were obtained using inks with increasing graphene concentration. The graphene ink on sensor field S_4_ resulted in a strong coffee stain due to a small misalignment of the print which caused a very low spreading of the ink. This sensor was used to investigate the difference in the gas sensitivity between a homogeneous graphene film and a sensor with strong coffee stain, thus a highly inhomogeneous graphene layer.

**Figure 2:**
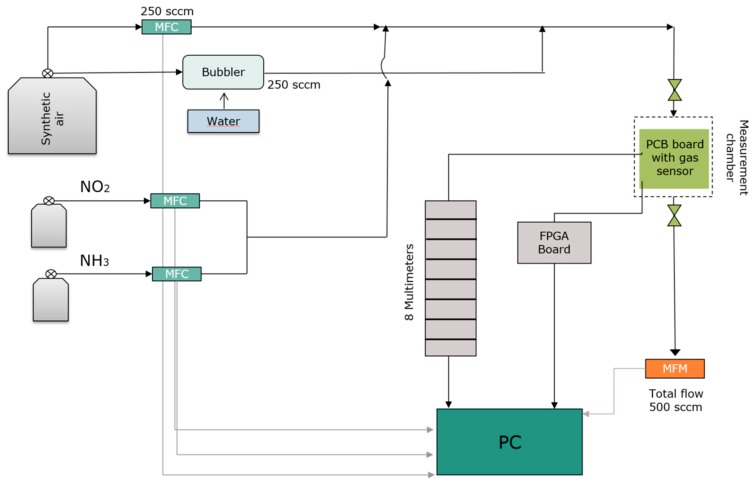
Schematic structure of the gas measuring station.

The setup, illustrated in Figure 2, employed test gases (NO_2_ and NH_3_) and synthetic air (20% O_2_ and 80% N_2_) as dilution gas. Additionally, it included a bubbler using 250 sccm of synthetic air as input flow and generating an output flow of humid air capable of producing a relative humidity up to 90%. Both NO_2_ and NH_3_ flows were provided by gas bottles produced by Linde with fixed concentrations of 100 ppm and 1000 ppm respectively, while their flow was regulated by a precision MFC (mass flow controller) subject to periodic calibration. The gas flows were therefore diluted by the synthetic dry air and by the humid air from the humidity generator, to reach the target gas concentration. The mixture of gas flows and synthetic air flow was kept constant at 500 sccm in order to keep a constant humidity in the total flow.

The MEMS heater located underneath the sensor was used for setting different sensor temperatures. The sensor heaters were controlled by an FPGA (Field Programmable Gate Array) and the sensor resistance were read by 8 multimeters (Agilent HP 3458A). The data were recorded in real time by the measuring software (MultiMess7) installed on the computer. The MFCs (mass flow controllers), the bubbler, and the heater temperature were regulated using a Visual Studio Macro. A steel chamber was used for the measurements to reduce the adsorption of water or gases.

## 3. Results

**Figure 3:**
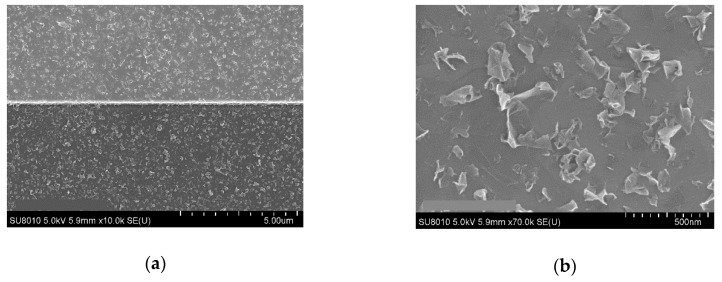
SEM picture of the graphene flakes after drying the ink: (**a**) Low magnification picture; (**b**) High magnification picture.

**Figure 4:**
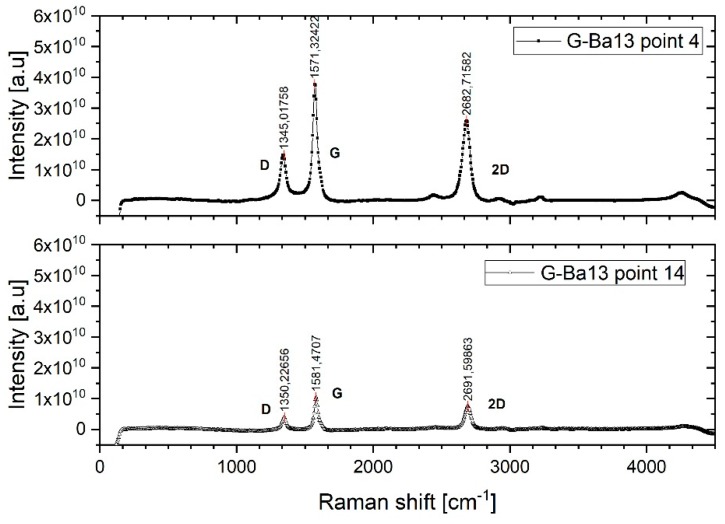
Raman spectroscopy of the graphene flakes after thermal post treatment of the printed ink, using a laser excitation of 532 nm.

The graphene layer was investigated after printing and drying, using Raman spectroscopy and SEM. SEM analysis shows that part of the graphene flakes crumbles on the surface while the other part is flat and adhering on the substrate with their whole area, as can be seen in Figure 3. This might be due to the method used to produce them or due to their small lateral size. The SEM picture shows that flakes have different lateral dimension, while most of them are in the range of 100–500 nm. Statistical investigation of the flakes done by the supplier, measuring 1000 flakes under SEM, showed that the average lateral size is about 500 nm. The thickness of the flakes was measured by the producer and is of few nm. However, since these flakes are crumpling on the surface, the thickness of a film of flakes produced using graphene ink with concentration of 1 g/L measured using AFM, showed values of 5 nm or higher in the presence of some small agglomerations of flakes, as can be observed in Figure 4.

Raman spectroscopy is a technique largely used to investigate the chemical and physical properties of graphene; it can be used for studying thickness, disorder, edge, and grain boundaries, doping and thermal conductivity [27,28,29,30]. The main Raman peaks used to characterize graphene are: The *G* peak, which is a primary in-plane vibration mode and the 2*D* peak, which is a double phonon scattering (second-order overtone of a different in-plane vibration). Other three peaks can appear in the spectrum: *D* (at 1350 cm^−1^ from a 532 nm excitation laser), *D*’ (at 1620 cm^−1^ from a 532 nm excitation laser), and *D* + *D*’ (at 2940 cm^−1^ from a 532 nm excitation laser). The *D* peak is caused by intervalley phonons (scatters from *K* to *K*’) and defect scattering and it is not visible in pristine graphene due to crystal symmetries, since it needs a defect to cause a second elastic scattering. The *D* peak increases with the amount of defects but it is not dependent on their geometry. The *D*’ peak is due to intravalley phonons (scatters from *K* to *K*’) and defect scattering. Its level depends on the type of defect, i.e., is more sensitive to vacancies than *sp*^3^ sites. The *sp*^3^ sites introduce a different arrangement of the carbon atoms, but do not break the network [27,30,31,32]. This peak is always present in defects in graphene, even if it is often difficult to measure due to the superposition with the *G* peak [33,34]. The *D* + *D*’ is also a defect activated peak and is only visible in the presence of high amount of defects [12,18].

Figure 4 shows Raman spectra of the graphene flakes used, acquired under different positions of the graphene film. It can be observed that the ratio between the peaks is similar in the two spots. The different intensity is due to the different amount of flakes in the two areas. The *D* peak indicates the presence of point and edge defects. The *D* peak is low and sharp and lower than the *G* peak indicating a relatively low amount of defects in the material. In the case of nanocrystallite graphite (low defect density regime), *I_D_*\*I_G_* will increase with the increasing amount of defects due to the higher elastic scattering. In the case of amorphous carbon (high defect density regime), the lower the *I_D_*\*I_G_*, the smaller the inter-defect distance, since the defect density in this structure is so high that all the Raman peaks are attenuated [30,31,35]. The *I_D_*\*I_G_* of the graphene flakes investigated is about 0.6, which indicates an average crystallite size of 13 nm [13].

The number of layers can be estimated from the shape and position of the 2*D* peak. In few-layer graphene, compared to monolayer graphene, the intensity of the 2*D* peak decreases and the peak broadens and shifts to lower frequency [30,35,36]. The shape of the 2*D* peak would suggest that the material consists of few-layer thickness graphene platelets. No *D*’ modes are visible due to the superposition of the *G* and *D*’ modes, but it is always present in graphene with defects and can be estimated as half of the 2*D*’ peak (at about 3160 cm^−1^ at 2.33 eV) [33,37]. As can be seen from the graphs of Raman spectra, the intensity values of this mode is quite weak, indicating the presence of a small *D*’ peak superposed by the *G* peak. Since *D*’ is more sensitive to vacancies compared to *sp*^3^ sites, this means that the majority of the defects in this material are *sp*^3^ hybridizations rather than vacancies [30].

**Figure 5:**
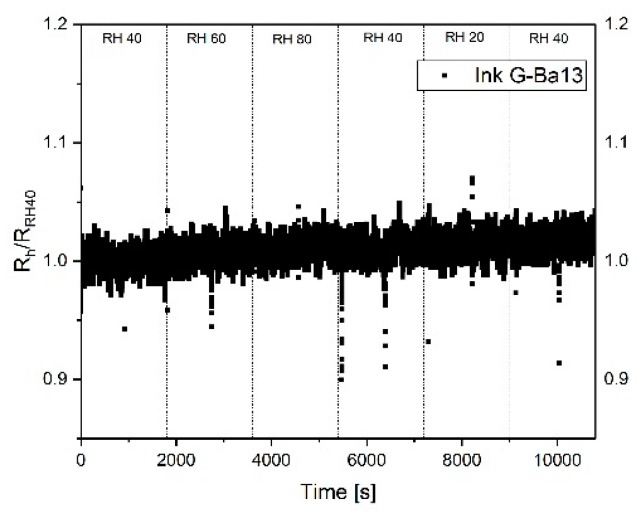
Normalized response of sensor S_2_ towards different relative humidity. The sensor was heated up to 250 °C and kept at this temperature during the measurements.

Figure 5 shows the response of the sensor S_2_, printed using a graphene concentration of 1 g/L towards humidity. The sensor was heated up at 250 °C directly before starting the measurements and kept at this temperature during the measurements; this procedure helps remove the water molecules from the surface. All produced sensors do not exhibit a strong change in the resistance with respect to relative humidity. This represents a significant advantage to MOX sensors. The motivation for this behavior is not yet fully understood. One hypothesis is that the resistance change due to adsorption of water molecules at the boundary defects is partially compensated by the edge defects, where molecules form conductive chains through the graphene flakes [37]. A second hypothesis sees the sensor’s surface hydrophobicity reduce the probability of water adsorption, leading to a reduced sensitivity to humidity [38].

**Figure 6:**
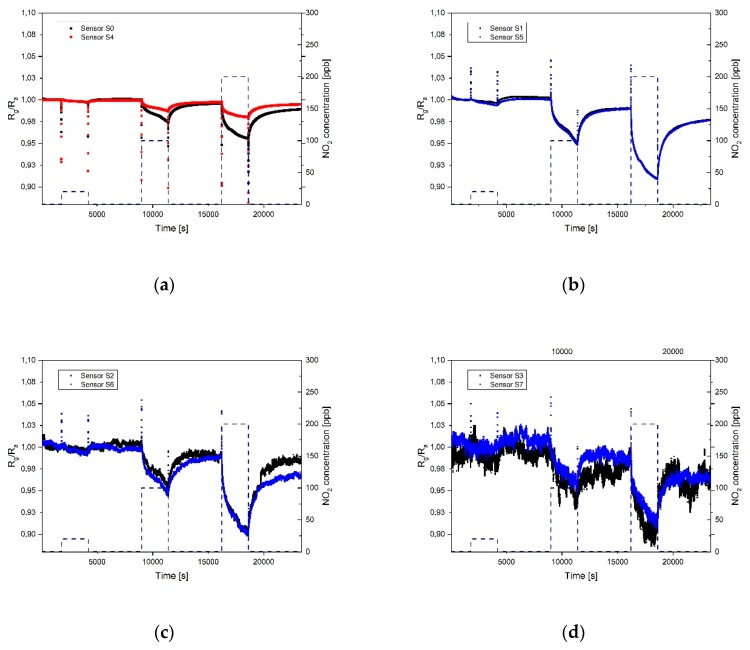
Dynamic response of the sensors towards different concentrations of NO_2_ (20, 100, and 200 ppb): (**a**) Sensors S_0_ and S_4_; (**b**) sensors S_1_ and S_5_; (**c**) sensors S_2_ and S_6_; (**d**) sensors S_3_ and S_7_. The temperature of the heater underneath the sensor was 250 °C and the relative humidity in the test cell was 20% (at room temperature).

**Figure 7:**
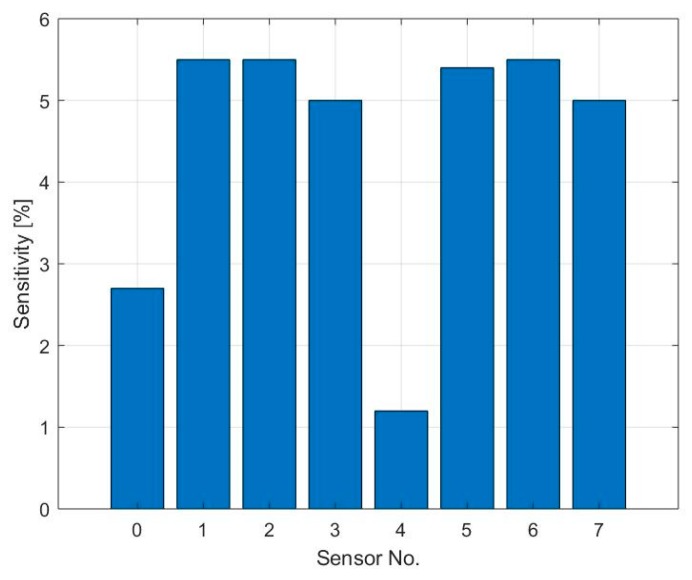
Summary of the sensitivity towards 100 ppb of NO_2_ of the 8 sensors.

The response of the different sensor fields towards NO_2_ and NH_3_ were measured keeping the relative humidity of the synthetic air and gases mixture to a value of 20%, which represent a dry ambient environment. In Figure 6 the response of the 8 sensors is shown with respect to three different concentrations of nitrogen dioxide: 20 ppb, 100 ppb, and 200 ppb. Heating was applied to the sensor during measurements with the twofold aim to remove water molecules from the sensor surface and provide the energy needed to enhance the adsorption of gases on the graphene layer during exposure and desorption during the cleaning phase under synthetic air. Sensor S_0_ has a thick graphene layer, homogeneously distributed over the electrode surface, and its response to the different concentrations of gases is represented by the black curve in Figure 6a. Sensor S_4_ has the same amount of graphene flakes, but a strong coffee stain, which means that the sensing area is drastically reduced compared to S_0_. Its response is represented by the red curve. The response of sensor S_0_ is faster and shows a higher sensitivity compared to S_4_, as expected due to the smaller sensing area. The recovery of sensor **S**_0_ is slower due to the higher adsorption of the gas molecules. The fast response of the sensor can be explained by an initial physisorption mechanism. The long recovery time is caused by the presence of both physisorption and chemiadsorption phenomena; the defects in the graphene flakes indeed cause, most likely, chemiadsorpion on the defect sites [31]. It can be observed in Figure 6 and Figure 7 that the sensitivity initially increases using a thinner sensing layer, due to the fact that the electrical conduction takes place on the surface and it is therefore directly influenced by the adsorption of even a few molecules. On the other side, when the layer is thick, the conduction of the sensor is not strongly influenced by the interaction of the gas molecules since in multilayer graphene the current flows through independent parallel conduction paths, so part of the carrier transport is happening in the underlying graphene layer which does not interact with the gas [32]. Nevertheless, when then the layer is too thin, like for sensors S_3_ and S_7_, probably also due to an insufficient amount of material to form a continuous film, the sensor resistance is high and a high thermal noise level can be observed on the output signal. In this case, a deviation in the sensitivity of similar sensors is observed, caused by the noise and the smaller sensing area. In all the presented measurements of the 8 sensor fields, the signal during adsorption did not reach the saturation.

During exposure to NO_2_ the sensors exhibit a first rapid response, which corresponds to the first steep part of the slope, followed by a slower response which corresponds to a shallower slope. According to Robinson et al., the rapid response comes from the adsorption of gas molecules into low energy binding sites, like *sp^2^* bonded carbon, while the slow response is caused by the gas molecular interaction with higher energy binding sites, e.g., vacancies, structural defects, and oxygen functional groups. The adsorption of molecules into low energy binding sites occurs through physisorption and involves weak dispersive forces, while the interaction with higher energy binding sites involves a chemisorption mechanism and therefore energies of several hundred meV/molecule. The physisorption which occurs during the rapid response is therefore recoverable, while the chemisorption which occurs during the slow response needs some moderate heating to recover [36,39].

**Figure 8:**
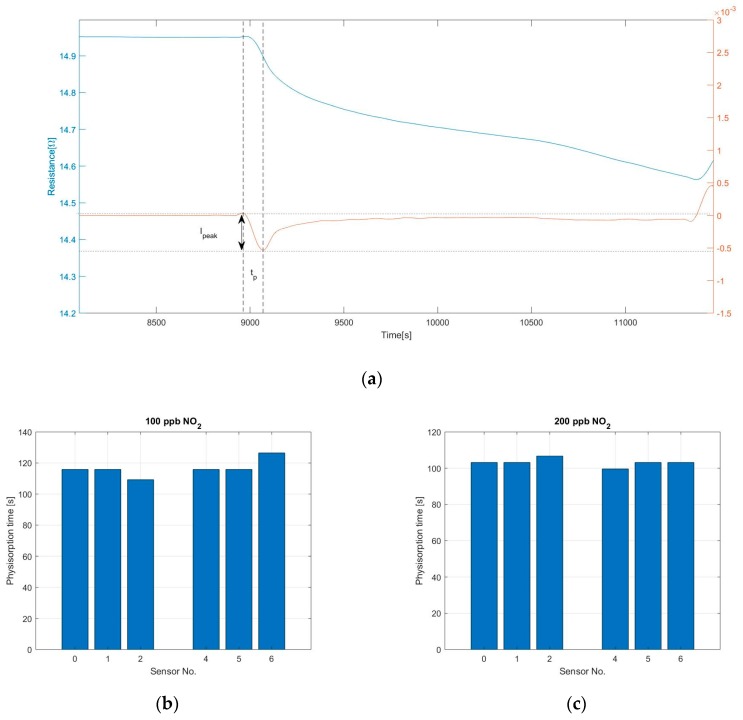

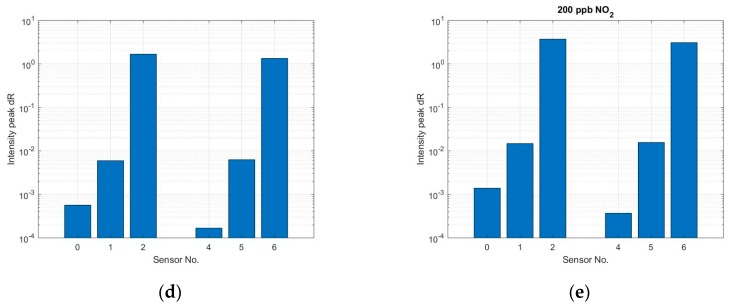
(**a**) The blue curve shows the response of sensor S_0_ after exposure to 100 ppb of nitrogen dioxide, the red curve shows the derivative of the same sensor. The temperature of the sensor during the measurement was 250 °C and the relative humidity inside the test cell was 20%; (**b**,**c**) Physisorption time of sensors S_0_, S_1_, S_2_, S_4_, S_5,_ and S_6_ when exposed to 100 and 200 ppb NO_2_, calculated using the first derivative; (**d**,**e**) Peak intensity of the first derivative of the sensor’s signal during the physisorption. This value was used to compare the response speed of the different sensors.

The response time of the sensors cannot be properly calculated since the signal did not reach saturation, however, the physisorption time has been estimated through an analysis of the first derivative, as shown in Figure 8a. Due to the high level of noise at the output signal of sensors S_3_ and S_7_ and the small reaction at low gas concentration, this analysis is presented just for 6 of the sensors and for NO_2_ concentration of 100 and 200 ppb. It can be observed in Figure 8b,c that the physisorption time is similar for all the sensors analyzed and it is around 115 s when exposed to 100 ppb NO_2_ and 100 s when at 200 ppb NO_2_, caused by a faster occupation of low energy binding sites when more gas molecules are present. The peak amplitude of the signal’s first derivative has been used to compare the reaction time of the different sensors, this provides a quantitative value for the maximum steepness of the sensor’s initial response. Results are visible in Figure 8d,e. As previously assumed, the reaction is faster for the sensor with thinner graphene film, while the sensor with strong coffee stain shows a slower response compared to the sensors printed with the same ink but with a more homogeneous layer.

**Figure 9:**
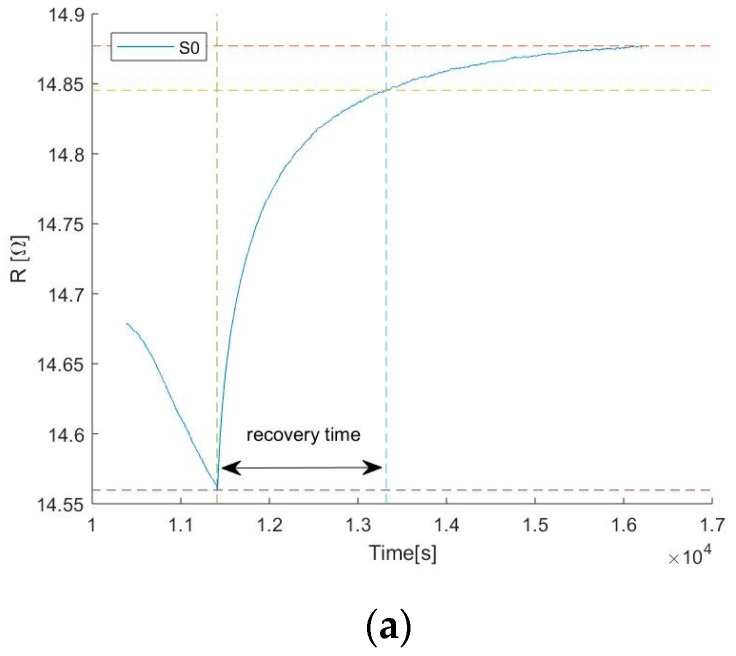

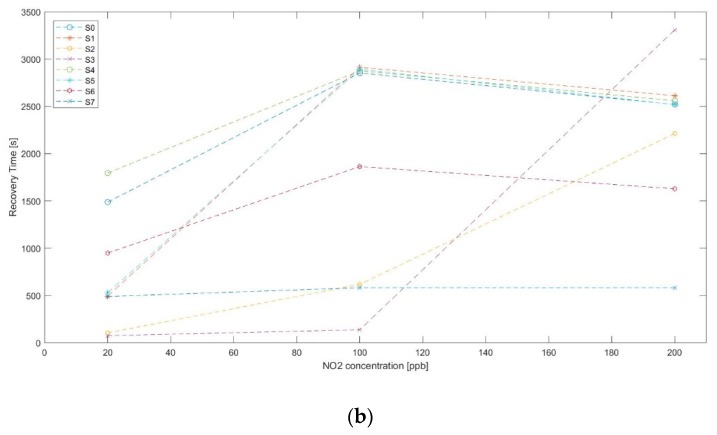
(**a**) The graph shows the recovery of sensor S_0_ after exposure to 100 ppb NO_2_ while keeping the heater at a constant temperature of 250 °C. The sensor’s recovery time has been calculated as the time until the sensor’s output reaches the 90% of its stable recovered condition; (**b**) Recovery time of sensors S_0_, S_1_, S_2_, S_3_, S_4_, S_5,_ S_6_ and S_7_ after exposure to different concentrations of NO_2_.

The sensor’s recovery time has been calculated as the time for the sensor to reach the 90% of its stable recovered condition, as displayed in Figure 9a,b illustrates the recovery time of sensors S_0_, S_1_, S_2_, S_3_, S_4_, S_5_, S_6,_ and S_7_ after exposure to different concentrations of NO2. Sensors S_0_ and S_4_, which have higher amounts of graphene flakes show a very slow recovery, 30 min for desorbing 20 ppb NO_2_ and more than 40 min for desorbing 100 and 200 ppb NO_2_. Sensors S_1_ and S_5_ show three times faster recovery from 20 ppb NO_2_ exposure, while the recovery from higher gas concentration is very similar to S_0_ and S_4_. The other sensors show a noisier signal and therefore different response times even with similar amounts of graphene. However, the results indicate that thinner graphene layers have faster recovery time. The hypothesis is that the thick graphene layer presents a porous structure due to the stacking of crumbled graphene flakes which can slow down the desorption of the gas molecules from the film.

**Figure 10:**
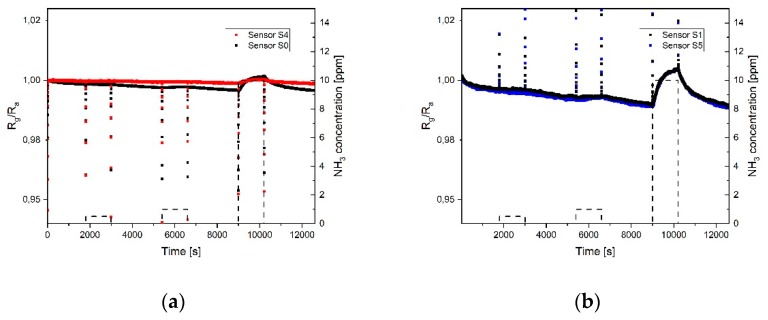

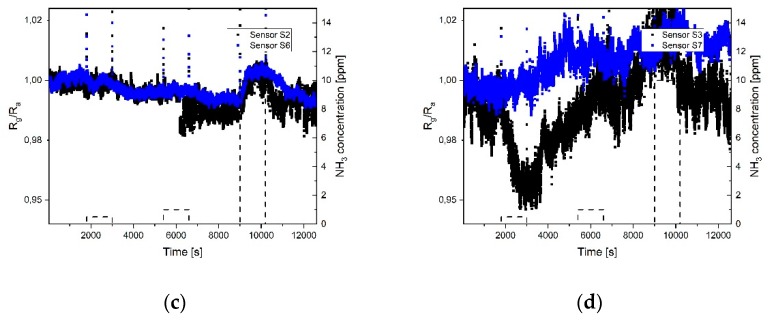
Dynamic response of the sensors toward different concentrations of NH_3_ (0.5, 1, 10 ppm): (**a**) Sensors S_0_ and S_4_; (**b**) sensors S_1_ and S_5_; (**c**) sensors S_2_ and S_6_; (**d**) sensors S_3_ and S_7_. The temperature of the heater underneath the sensor was 250 °C and the relative humidity in the test cell was 20% (at room temperature).

**Figure 11:**
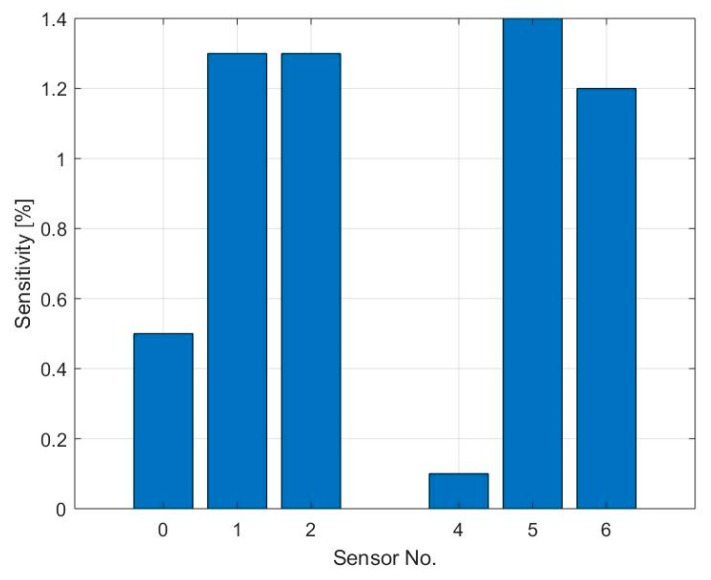
Summary of the sensitivity towards 10 ppm of NH_3_ of sensors S_0_, S_1_, S_2_, S_4_, S_5,_ and S_6_.

Figure 10 and Figure 11 show the response and sensitivity of the different sensor fields towards 0.5, 1, and 10 ppm ammonia in an environment with 20% relative humidity and using a heater temperature of 250 °C. As expected, and as already shown with nitrogen dioxide, the sensor with coffee stain (S_4_) has a very low sensitivity and almost no reaction at 0.5 and 1 ppm NH_3_. Sensor S_0_ shows response even to the lower concentration but also a drift in the baseline, due to an incomplete desorption. In Figure 10b the response of sensors S_1_ and S_5_ is plotted, showing the same response for the two sensors. It can be observed that at low NH_3_ concentration the slope of the response is shallow, indicating that the adsorption centers with high binding energy are occupied first, this also explains that almost no recovery happens at low gas concentration [36].

At the higher NH_3_ concentration (10 ppm) a fast response occurs, followed by a slower one, which would suggest physisorption on the low binding energy adsorption centers and just after this phase the occupation of adsorption centers with high binding energy. In this case a fast recovery of the sensor is observed. As visible in Figure 10c the response of sensors S_2_ and S_6_ is noisy and a response towards NH_3_ can be detected just at high concentration. Sensors S_3_ and S_7_ (Figure 10d) show too much thermal noise due to high resistance and most probably the reduced sensing area, therefore do not provide any useful information. 

The sensitivity of these sensors towards ammonia is significantly lower than the one towards nitrogen dioxide due to the lower binding energy which causes a lower adsorption density [40,41,42].

**Figure 12:**
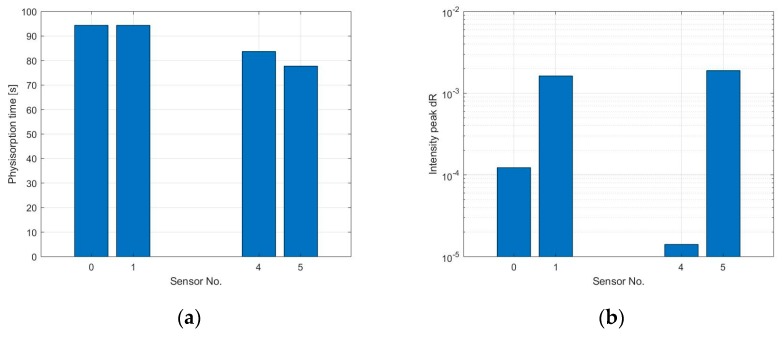
(**a**) Physisorption time of sensors S_0_, S_1_, S_4_, and S_5_ when exposed to 10 ppm NH3, calculated using the first derivative approach; (**b**) Intensity of the peak of the first derivative of the sensor’s signal during the physisorption. This value has been used to compare the response speed of the different sensors.

The physisorption time and the first derivative’s peak amplitude have been calculated only for sensors S_0_, S_1_, S_4_, and S_5_ and during exposure to 10 ppm NH_3_ due to the low signal to noise ratio and very low response of the other sensors at lower concentrations. Figure 12a shows the physisorption time, which is between 80 and 95 s. The intensity of the peak of the first derivative is higher for sensors S_1_ and S_5_ compared to the sensors with higher amount of graphene, indicating a stronger response of the sensors, which is consistent with the sensor’s sensitivity (displayed in Figure 11).

**Figure 13:**
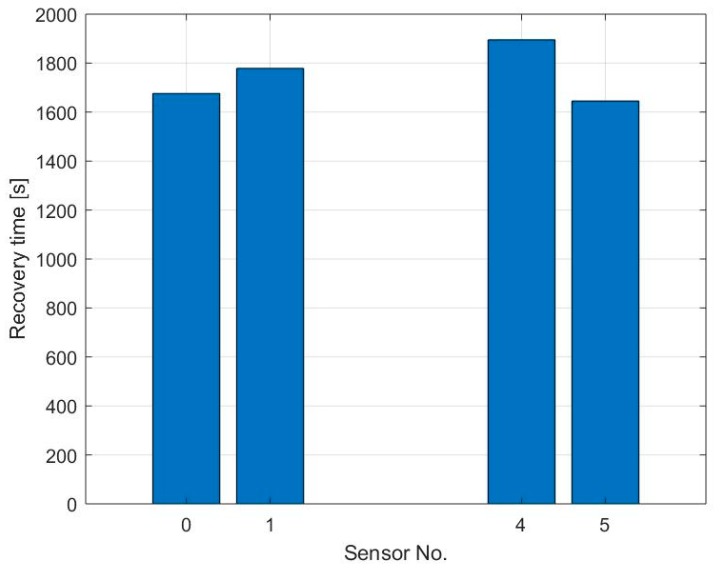
The graph shows the recovery time of sensors S_0_, S_1_, S_4,_ and S_5_ after exposure to 10 ppm NH_3_ while keeping the heater at a constant temperature of 250 °C. The sensor’s recovery time has been calculated as the time until the sensor’s output reaches the 90% of its stable recovered condition.

The recovery time of sensors S_0_, S_1_, S_4,_ and S_5_ after exposure to 10 ppm NH_3_ is illustrated in Figure 13. All analyzed sensors show a recovery time higher than 30 min, while the sensor with coffee stain shows the highest recovery time.

**Figure 14:**
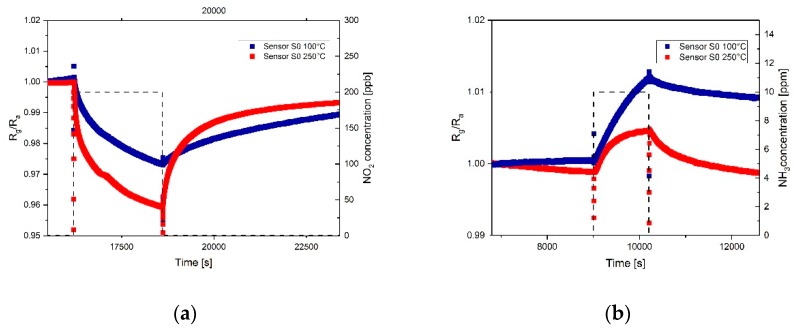
(**a**) Response of the sensors S_0_ towards 200 ppb of NO_2_. The red curve shows the response of the sensor during measurements at 250 °C; the blue curve shows the response of the sensor during measurements at 100 °C; (**b**) Response of the sensors S_0_ towards 10 ppm of NH_3_. The red curve shows the response of the sensor during measurements at 250 °C; the blue curve shows the response of the sensor during measurements at 100 °C.

The response of sensor S_0_ towards 200 ppb NO_2_ and 10 ppm NH_3_ at two different temperatures of the sensor, 100 and 250 °C, is shown in Figure 14. The response of the sensor towards nitrogen dioxide at 100 °C is weaker compared to the measurements at 250 °C, due to the lower amount of thermal energy provided to the molecules during adsorption. The recovery of the sensor is also faster at 250 °C since the higher temperature provides energy which helps desorbing the gas molecules from the graphene layer. These results are consistent with the ones reported by Fowler et al [43]. As can be observed in Figure 14b, the sensitivity towards ammonia shows an opposite trend, it is higher at lower temperature. The lower sensitivity of the sensors towards NH_3_ at higher temperature might be caused by thermal fluctuations of the NH3 molecules which decrease the probability of the molecules to attach on the surface [44]. Since the heat capability of gases is higher for lower molecular weight [45] and ammonia’s molecular weight is three times lower than the one of NO_2_, this would cause higher thermal fluctuations in the presence of ammonia rather than nitrogen dioxide. Therefore, the operation temperature of the sensor should be optimized if higher sensitivity towards ammonia is required.

**Figure 15:**
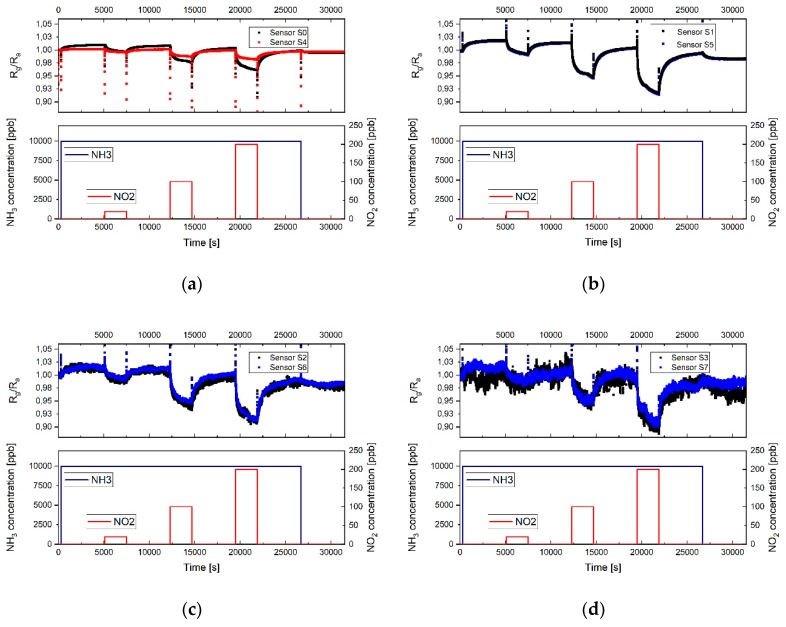
Dynamic response of the sensors towards 10 ppm NH_3_ and different concentrations of NO_2_: (**a**) Sensors S_0_ and S_4_; (**b**) sensors S_1_ and S_5_; (**c**) sensors S_2_ and S_6_; (**d**) sensors S_3_ and S_7_. The temperature of the heater underneath the sensor was 250 °C and the relative humidity in the test cell was 20% (at room temperature).

Figure 15 shows the results of cross sensitivity measurements run at 20 % RH and 250 °C conditions using 10 ppm of NH_3_ and different concentrations of NO_2_. As expected some cross sensitivity can be observed between NO_2_ and NH_3_ due to the reaction of the sensor to both gases. Since ammonia is donating electrons while nitrogen dioxide is accepting them and behaving graphene as a *p*-type material, the first gas will increase the resistance of the sensor and the second will decrease it. This would lead to an underestimation of the NO_2_ concentration compared to the real value and a non-detection of NH_3_. However, since the sensitivity of the sensor towards ammonia is significantly lower, if the ammonia concentration is below a few ppm this will not severely affect the sensitivity of the sensor.

## 4. Discussion

The presented sensors show a stable response when exposed to very different humidity levels, showing a great advantage compared to MOX. When the sensing layer is not homogeneous and does not cover all the electrode area, i.e., when coffee ring forms during ink drying, the reaction of the sensor towards gases is slower and so is the sensitivity due to the small sensing area and thus reduced number of adsorption sites. The right trade-off in the sensing film thickness is important: On one side when the layer is too thick the current flows through independent parallel paths, which brings to a lower sensitivity, on the other side when the film is too thin, thermal noise degrades the signal to noise ratio at the output. Moreover, thinner graphene films show higher sensitivity and faster recovery time. The sensitivity of the sensor towards ammonia is lower than the one towards NO_2_ because of the lower adsorption density and the operation temperature of the sensor being optimized for nitrogen dioxide [16,42]. At low NH_3_ concentration the adsorption seems to be dominated by chemisorption and this would explain the slow desorption, while at higher concentration physisorption also contributes and desorption occurs faster. Cross sensitivity measurements at high NH_3_ concentration have shown little influence compared to NO_2_, however, to be able to distinguish and predict the concentration of two different gases, employing more sensors with differently doped graphene becomes necessary. Some drift in the baseline has been observed but this is due to incomplete desorption and can be reduced by introducing higher temperature pulses to enhance the gas desorption or can be compensate by periodic calibration and data post processing
